# Melatonin stimulates aromatase expression and estradiol production in human granulosa-lutein cells: relevance for high serum estradiol levels in patients with ovarian hyperstimulation syndrome

**DOI:** 10.1038/s12276-020-00491-w

**Published:** 2020-08-27

**Authors:** Jung-Chien Cheng, Lanlan Fang, Yiran Li, Sijia Wang, Yuxi Li, Yang Yan, Qiongqiong Jia, Ze Wu, Zhen Wang, Xiaoyu Han, Ying-Pu Sun

**Affiliations:** Center for Reproductive Medicine, Henan Key Laboratory of Reproduction and Genetics, The First Affiliated Hospital of Zhengzhou University, 450052 Zhengzhou, China

**Keywords:** Endocrine reproductive disorders, Endocrine reproductive disorders

## Abstract

Ovarian hyperstimulation syndrome (OHSS) is one of the most life-threatening and potentially fatal complications associated with controlled ovarian hyperstimulation (COH) during in vitro fertilization (IVF) treatment. Although the pathogenesis of OHSS remains unclear, elevated serum estradiol (E2) levels before human chorionic gonadotropin (hCG) administration are associated with the risk of OHSS. The pineal hormone melatonin and its receptors are expressed in human granulosa cells and have been shown to stimulate E2 production. However, the effect of melatonin on the expression of aromatase, an enzyme responsible for a key step in the biosynthesis of E2, in human granulosa cells remains to be determined. Here, we demonstrate that melatonin upregulates aromatase expression in primary cultured human granulosa-lutein (hGL) cells through the melatonin receptor-mediated PKA-CREB pathway. Using a mouse model of OHSS, we demonstrate that administration of the melatonin receptor inhibitor luzindole inhibits the development of OHSS. In addition, the expression of ovarian aromatase and serum E2 levels are upregulated in OHSS mice compared to control mice, but this upregulation is attenuated by inhibition of the function of melatonin. Moreover, clinical results reveal that aromatase expression levels are upregulated in hGL cells from OHSS patients. Melatonin and E2 levels in the follicular fluid are significantly higher in OHSS patients than in non-OHSS patients. Furthermore, melatonin levels are positively correlated with E2 levels in follicular fluid. This study helps to elucidate the mechanisms mediating the expression of aromatase in hGL cells and provides a potential mechanism explaining the high E2 levels in patients with OHSS.

## Reproductive medicine: Melatonin implicated in complication of assisted fertilization

Blocking the activity of melatonin helps prevent female mice from developing swollen ovaries, a potentially life-threatening complication of assisted fertilization techniques. A research team from China’s First Affiliated Hospital of Zhengzhou University, led by Jung-Chien Cheng and Ying-Pu Sun, demonstrated that melatonin, a hormone found in the fluid that surrounds developing eggs, can induce ovarian cells to boost expression of aromatase. This enzyme is responsible for synthesizing estradiol, a hormone involved in female reproduction. Women whose ovaries became over-stimulated in response to fertility medications showed elevated levels of melatonin, aromatase and estradiol. Inhibiting the function of melatonin reduced symptoms in mouse models of ovarian hyperstimulation syndrome. The findings reveal an important role of melatonin in ovarian enlargement and point to new therapeutic strategies for women undergoing in vitro fertilization.

## Introduction

Estradiol (E2), the most active form of natural estrogens, is a steroid hormone synthesized from ovarian granulosa cells that plays an important role in female reproduction. Blocking E2 synthesis or actions results in severe reproductive defects^1^. Various physiological functions of the ovary, including folliculogenesis, ovulation, luteal function, and steroidogenesis, are regulated by E2. The biosynthesis of E2 is catalyzed by aromatase, which is encoded by the cytochrome P450 family 19 subfamily A member 1 (*CYP19A1*) gene. It is known that aromatase is expressed in granulosa cells of ovarian preovulatory follicles. After ovulation, granulosa cells differentiate into granulosa-lutein (hGL) cells that express aromatase and retain the capacity to synthesize E2^2,3^. In human granulosa cells, pituitary secreted follicle-stimulating hormone (FSH) has been identified as the most important factor that regulates the expression of aromatase^3^. However, several growth factors and hormones have also been reported to regulate aromatase expression in granulosa cells.

In assisted reproductive technology, controlled ovarian hyperstimulation (COH) is usually performed to produce more oocytes. Ovarian hyperstimulation syndrome (OHSS) is a serious complication associated with COH. During COH, OHSS occurs after gonadotropin stimulation followed by human chorionic gonadotropin (hCG) administration. The risk of severe OHSS in an unselected population is ~2%; however, the incidence may be as high as 20% among high-risk patients. The symptoms of OHSS include enlarged ovaries, massive fluid shift and accumulation in the peritoneal cavity and other organs, renal failure and venous embolism. Although OHSS is considered as an iatrogenic complication, it may sometimes lead to maternal death^4–6^. OHSS theoretically could occur in any woman undergoing COH. However, some known risk factors for the development of OHSS have been identified, such as age <30 years, body mass index (BMI) < 20 and polycystic ovarian syndrome (PCOS). Interestingly, high serum E2 levels before hCG administration (>3500 pg/mL) are significantly associated with the development of OHSS^7,8^. It is known that a high ovarian follicle count may contribute to high serum E2 levels in OHSS. However, whether the expression of aromatase varies between non-OHSS and OHSS patients has not been determined.

Melatonin is a hormone synthesized and released at night by the pineal gland that regulates the sleep-wake cycle, pubertal development, and seasonal adaptation^9,10^. For many years, melatonin was considered to be a hormone produced only by the pineal gland. Following the development of highly sensitive antibodies against melatonin, it was shown that this hormone can be produced by many extrapineal tissues and exerts its biological functions locally^11^. While it is known that many hormones are synthesized and produced by multiple cell types, none of these hormones appear to have the same broad spectrum of sources as melatonin. These findings suggest that melatonin plays important roles in the regulation of various physiological functions via endocrine, paracrine and autocrine mechanisms. In humans, melatonin is expressed in female reproductive organs, such as the ovary, placenta, and uterus^12^. In the ovary, melatonin is presented in the follicular fluid. The levels of melatonin in follicular fluid are positively correlated with the size of the follicle^13^. Interestingly, the levels of melatonin in follicular fluid are significantly higher than those in serum^14,15^, suggesting that melatonin is related to the regulation of human follicular function.

Melatonin receptors, namely, MT1 (*MTNR1A*) and MT2 (*MTNR1B*), belong to the G-protein-coupled receptor superfamily of membrane receptors^16^. Both MT1 and MT2 are expressed in hGL cells^17^. Previous studies in human and porcine granulosa cells have shown that melatonin treatment stimulates E2 production^18–20^. However, the effect of melatonin on aromatase expression in human granulosa cells remains unclear. Our previous study demonstrated that MT2 expression is upregulated in granulosa cells from OHSS patients and that melatonin levels in follicular fluid of OHSS patients are significantly higher than those in follicular fluid of non-OHSS patients^15^. Given the critical role of E2 in the development of OHSS, we hypothesized that high levels of melatonin in follicular fluid contribute to the pathogenesis of OHSS by upregulating aromatase expression and E2 production. In the present study, we examined the effect and the underlying molecular mechanisms of melatonin on aromatase expression in hGL cells. We also explored the role of melatonin in the pathogenesis of OHSS in mice and examined the expression of aromatase in hGL cells from OHSS patients.

## Materials and methods

### Antibodies and reagents

A monoclonal anti-aromatase antibody was obtained from Bio-Rad Laboratories (#MCA2077) (Shanghai, China). A monoclonal anti-α-tubulin antibody was obtained from Engibody (#AT0007) (Shanghai, China). Monoclonal anti-phospho-CREB (Ser^133^) (#9198) and anti-CREB (#9197) antibodies were obtained from Cell Signaling Technology (Shanghai, China). Horseradish peroxidase-conjugated goat anti-rabbit and goat anti-mouse IgG antibodies were obtained from Bio-Rad Laboratories. Melatonin, luzindole and H-89 were obtained from Sigma–Aldrich Corp. (Shanghai, China).

### Human follicular fluid samples

The study received approval and was carried out in accordance with the approved guidelines of the Zhengzhou University Research Ethics Board. Written informed consent was obtained from all patients before clinical samples were collected. None of the women had been prescribed any medications before enrollment. Human follicular fluid samples were obtained from 60 women (40 non-OHSS patients and 20 OHSS patients) during in vitro fertilization (IVF) treatment. All patients were between the ages of 20 and 35 and had normal menstrual cycles (Supplemental Table 1). Their causes of infertility were tubal obstruction or male infertility. Patients with polycystic ovarian syndrome, endometriosis, diminished ovarian reserve, chromosome abnormality or hydrosalpinx were excluded from this study. All patients were treated with a standard long protocol. During the mid-luteal phase, the gonadotropin-releasing hormone (GnRH) agonist triptorelin (0.1 mg) (Ipsen Pharma Biotech, France) was administered subcutaneously daily. Approximately 14 days after the injection of GnRH agonist, 150–300 IU recombinant FSH (Gonal-F; Merck, Germany) was administered daily. When at least three follicles had reached 18 mm, hCG (10000 IU, Livzon, Zhuhai, China) was injected. Oocyte retrieval was scheduled approximately 34–36 h after hCG injection by transvaginal ultrasound-guided follicular aspiration. Follicular fluid was collected when the oocytes were retrieved. Only the first follicular fluid aspirate without blood or flushing solution was used for analysis. After 10 min of centrifugation at 1200 rpm, the supernatant was stored at −80 °C until further analysis.

### Primary culture of human granulosa-lutein (hGL) cells

Primary hGL cells were purified from follicular aspirates collected from women undergoing oocyte retrieval as previously described^21^. Cells were cultured in a humidified atmosphere containing 5% CO_2_ and 95% air at 37 °C in Dulbecco’s modified Eagle’s medium/nutrient mixture F-12 Ham medium (DMEM/F-12; Gibco, Shanghai, China) supplemented with 10% charcoal/dextran-treated FBS (HyClone, Shanghai, China), 100 U/mL penicillin and 100 μg/mL streptomycin sulfate (Boster, Wuhan, China). For melatonin stimulation experiments, cells were cultured in 12-well plates at a density of 5 × 10^4^ cells/cm^2^ with 1 mL of culture medium for 5 days. All treatments were performed in medium containing 0.5% charcoal/dextran-treated FBS.

### Reverse transcription quantitative real-time PCR (RT-qPCR)

Total RNA was extracted with the RNeasy Plus Mini kit (QIAGEN, Shanghai, China) according to the manufacturer’s instructions. RNA (1 μg) was reverse-transcribed into first-strand cDNA with the iScript Reverse Transcription kit (Bio-Rad Laboratories). Each 20-μL qPCR reaction contained 1X SYBR Green PCR Master Mix (Applied Biosystems, Shanghai, China), 60 ng of cDNA and 250 nM of each specific primer. The primers used were 5′-GAG AAT TCA TGC GAG TCT GGA-3′ (sense) and 5′-CAT TAT GTG GAA CAT ACT TGA GGA CT-3′ (antisense) for human CYP19A1 aromatase; 5′-AAA CTT ACG TGG CTA CTC AGC ATC-3′ (sense) and 5′-GAC CTG GTT GAT GAT GCT CTT G-3′ (antisense) for human steroidogenic acute regulatory protein (StAR); 5′-CAG GAG GGG TGG ACA CGA C-3′ (sense) and 5′-AGG TTG CGT GCC ATC TCA TAC-3′ (antisense) for human P450 side chain cleavage enzyme (P450scc); 5′-GCC TTC CAG ACC AGA ATT GAG AGA-3′ (sense) and 5′-TCC TTC AAG TAC AGT CAG CTT GGT-3′ (antisense) for human 3β-hydroxysteroid dehydrogenase (3β-HSD); 5′-GAG TCA ACG GAT TTG GTC GT-3′ (sense) and 5′-GAC AAG CTT CCC GTT CTC AG -3′ (antisense) for human GAPDH; 5′-ACA CAT CAT GCT GGA CAC CT-3′ (sense) and 5′-GAG CTT GCC AGG CGT TAA AG-3′ (antisense) for mouse CYP19A1 aromatase; 5′-GAC CCT GGC TTT ACT GCT GT-3′ (sense) and 5′-AGA TGT CCA CCA GGG TCT CA-3′ (antisense) for mouse VEGF; 5′-TTG TGG AAG GGC TCA TGA-3′ (sense) and 5′-GAT GCA GGG ATG ATG TTC-3′ (antisense) for mouse GAPDH. qPCR was performed on an Applied Biosystems QuantStudio 12 K Flex system equipped with 96-well optical reaction plates. The specificity of each assay was validated by melting curve analysis and agarose gel electrophoresis of the PCR products. All of the RT-qPCR experiments were run in triplicate, and the mean values were used to determine mRNA levels. Water and mRNA without RT were used as negative controls. Relative quantification of mRNA levels was performed using the comparative Ct method with GAPDH as the reference gene using the formula 2^–∆∆Ct^.

### **Western blot**ting

Cells were lysed in cell lysis buffer (Cell Signaling Technology). Equal amounts of protein were separated by SDS polyacrylamide gel electrophoresis and transferred onto PVDF membranes. After 1 h of blocking with 5% nonfat dry milk in Tris-buffered saline (TBS), the membranes were incubated overnight at 4 °C with primary antibodies, which were diluted in 5% nonfat milk/TBS. Following primary antibody incubation, the membranes were incubated with the appropriate HRP-conjugated secondary antibody. Immunoreactive bands were detected using an enhanced chemiluminescent substrate (Bio-Rad Laboratories). The chemiluminescent blots were imaged with the ChemiDoc MP Imager (Bio-Rad Laboratories).

### Small interfering RNA (siRNA) transfection

To knock down endogenous CREB, cells were transfected with 100 nM ON-TARGETplus SMARTpool siRNA targeting the human CREB gene (Dharmacon, Lafayette, CO) using Lipofectamine RNAiMAX (Invitrogen). The siCONTROL NON-TARGETING pool siRNA (Dharmacon) was used as the transfection control.

### Measurement of melatonin and estradiol levels

Melatonin and estradiol levels in human follicular fluid and mouse serum samples or culture media were measured using an enzyme-linked immunosorbent assay (ELISA). A human melatonin ELISA kit (Abcam, Shanghai, China) and a human estradiol ELISA kit (Elabscience, Wuhan, China) were used in accordance with the manufacturer’s protocol. Estradiol levels in the culture media were normalized to the protein concentrations of the cell lysates. Estradiol levels in the culture media of treated cells were normalized to the levels in the culture media of control cells.

### Mouse OHSS model

Female ICR mice were obtained from Charles River Laboratories (Beijing, China). Animal handling was performed in accordance with the Guide for the Care and Use of Laboratory Animals published by the US National Institutes of Health. The mice were housed in an environmentally controlled room with free access to food and water. The animal studies were approved by the Zhengzhou University Animal Research Ethics Board. A mouse model of OHSS was established according to our previous study^22^. PMSG (20 IU/d) was administered i.p. to 5-week-old ICR female mice for 4 consecutive days, and hCG (7 IU, i.p.) was administered on the 4th day. Control mice were administered a single dose of PMSG (5 IU) followed by hCG (7 IU) 48 h later. Mice were treated with vehicle control (DMSO) or luzindole (10 mg/kg, i.p.) on days 4–6. All mice were euthanized on day 7. Each group contained eight mice. Changes in body weight and ovarian weight were recorded.

### Immunohistochemistry

Paraffin-embedded Section (5 μm) were deparaffinized and rehydrated. Antigen retrieval was conducted by boiling the sections in sodium citrate buffer (pH 6.0) for 8 min. Endogenous peroxidase activity was blocked by incubating the sections in 3% hydrogen peroxide solution at room temperature for 10 min. After 1 h of blocking with 3% bovine serum albumin in phosphate-buffered saline (PBS), the sections were incubated with a primary antibody against aromatase (#40809) (Signalway Antibody, Nanjing, China) overnight at 4 °C. Following primary antibody incubation, the sections were incubated with an HRP-conjugated secondary antibody. The sections were developed using the Peroxidase/DAB Dako REAL EnVision Detection System (#K5007) (Dako, Beijing, China) and counterstained with hematoxylin. A negative control sample that was not incubated with primary antibody was analyzed in parallel.

### Statistical analysis

The results are presented as the mean ± SEM of at least three independent experiments. All statistical analyses were analyzed by PRISM software (GraphPad Software, San Diego, CA). For experiments involving only two groups, the data were analyzed by *t*-test. Multiple comparisons were analyzed using one-way ANOVA followed by Tukey’s multiple comparison test. Significant differences were defined as *p* < 0.05.

## Results

### Melatonin stimulates aromatase expression in hGL cells

**Fig. 1:**
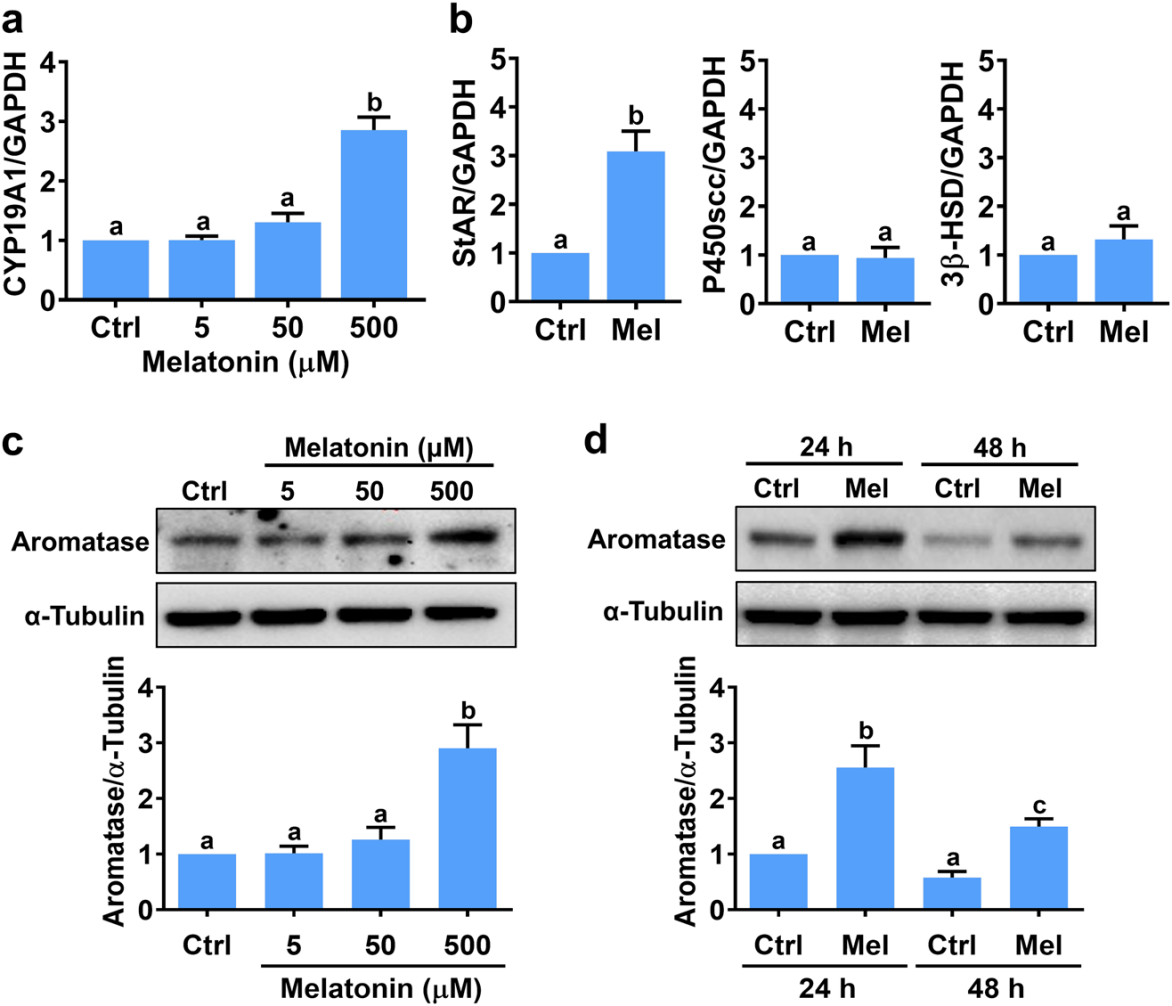
Melatonin stimulates aromatase expression in primary human granulosa-lutein cells. **a** Cells were treated with different concentrations of melatonin (Mel) for 24 h, and aromatase mRNA levels (CYP19A1) were examined by RT-qPCR. **b** Cells were treated with 500 µM melatonin (Mel) for 24 h, and the mRNA levels of StAR, P450scc, and 3β-HSD were examined by RT-qPCR. **c** Cells were treated with different concentrations of melatonin (Mel) for 24 h, and aromatase protein levels were examined by western blotting. **d** Cells were treated with 500 µM melatonin (Mel) for 24 or 48 h, and aromatase protein levels were examined by western blotting. The results are expressed as the mean ± SEM of at least three independent experiments. Values without a common letter are significantly different (*p* < 0.05).

To examine the effect of melatonin on aromatase expression, primary hGL cells isolated from the follicular aspirates of women undergoing oocyte retrieval during IVF treatment were treated with different concentrations of melatonin (5, 50, or 500 µM) for 24 h. While 5 or 50 µM melatonin had no significant effects, aromatase (*CYP19A1*) mRNA levels were significantly upregulated by exposure to 500 µM melatonin (Fig. 1a). Our recent study revealed that in hGL cells, melatonin stimulates the expression of steroidogenic acute regulatory protein (StAR), which is the key protein that mediates the rate-limiting step of ovarian steroidogenesis^23^. Consistent with our previous study, treatment of hGL cells with melatonin upregulated StAR mRNA levels, but the mRNA levels of two other steroidogenesis-related enzymes, P450 side chain cleavage enzyme (P450scc) and 3β-hydroxysteroid dehydrogenase (3β-HSD), were not affected by melatonin treatment (Fig. 1b). Western blotting confirmed the stimulatory effects of melatonin on aromatase at the protein level (Fig. 1c). In addition, time-course expression experiments showed that 24 h of melatonin treatment caused a significant upregulation of aromatase protein levels, and the stimulatory effect persisted 48 h after melatonin treatment (Fig. 1d).

### Stimulation of aromatase expression and E2 production by melatonin is mediated by MT1 and MT2 receptors

**Fig. 2:**
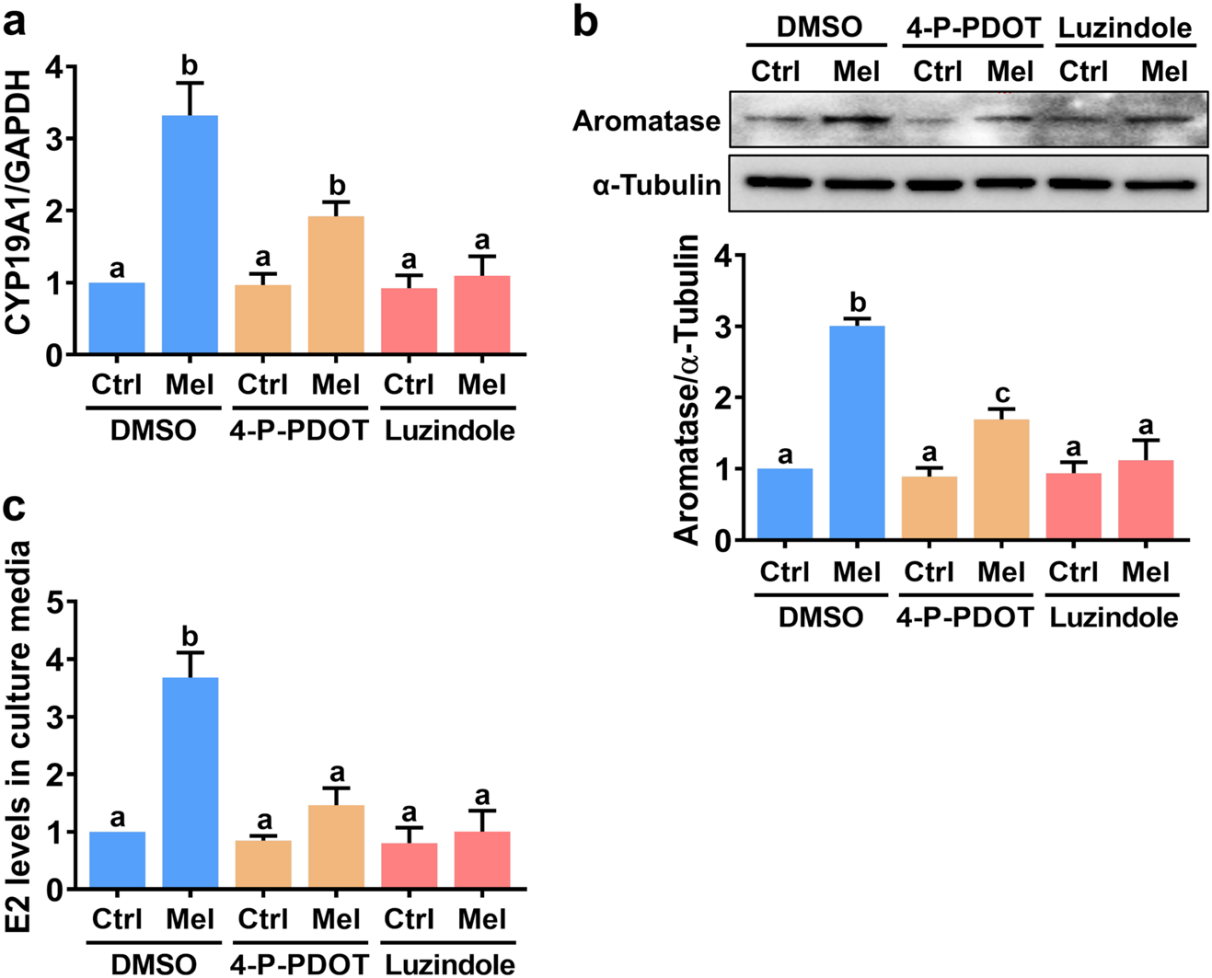
MT1 and MT2 melatonin receptors mediate melatonin-stimulated aromatase expression and E2 production in primary hGL cells. **a**, **b** Cells were pretreated with vehicle control (DMSO), 10 µM 4-P-PDOT, or 10 µM luzindole for 30 min and then exposed to 500 µM melatonin for 24 h. Aromatase mRNA (CYP19A1) (**a**) and protein (**b**) levels were examined by RT-qPCR and western blotting, respectively. **c** Cells were pretreated with vehicle control (DMSO), 10 µM 4-P-PDOT, or 10 µM luzindole for 30 min and then exposed to 500 µM melatonin for 24 h. The E2 levels in culture media were examined by ELISA. The results are expressed as the mean ± SEM of at least three independent experiments. Values without a common letter are significantly different (*p* < 0.05).

We next aimed to identify the cellular receptor that mediates the melatonin-induced upregulation of aromatase in hGL cells. To this end, two melatonin receptor antagonists, 4-P-PDOT (MT2-selective) and luzindole (MT1/MT2-nonselective), were used^24^. RT-qPCR and western blotting showed that neither of these antagonists affected basal aromatase mRNA or protein levels. Stimulation of aromatase mRNA and protein expression by melatonin was partially inhibited by pretreatment with 4-P-PDOT and abolished by pretreatment with luzindole (Fig. 2a, b). Given the vital role of aromatase in the regulation of E2 production, we examined the effect of melatonin on the production of E2 in hGL cells. ELISA showed that treatment of hGL cells with melatonin increased the release of E2 into the culture medium. In addition, melatonin-stimulated E2 production was blocked by inhibition of MT1 and MT2 (Fig. 2c). These results indicate that both MT1 and MT2 mediate melatonin-induced upregulation of aromatase expression in hGL cells.

### The PKA-CREB pathway mediates melatonin-stimulated aromatase expression

**Fig. 3:**
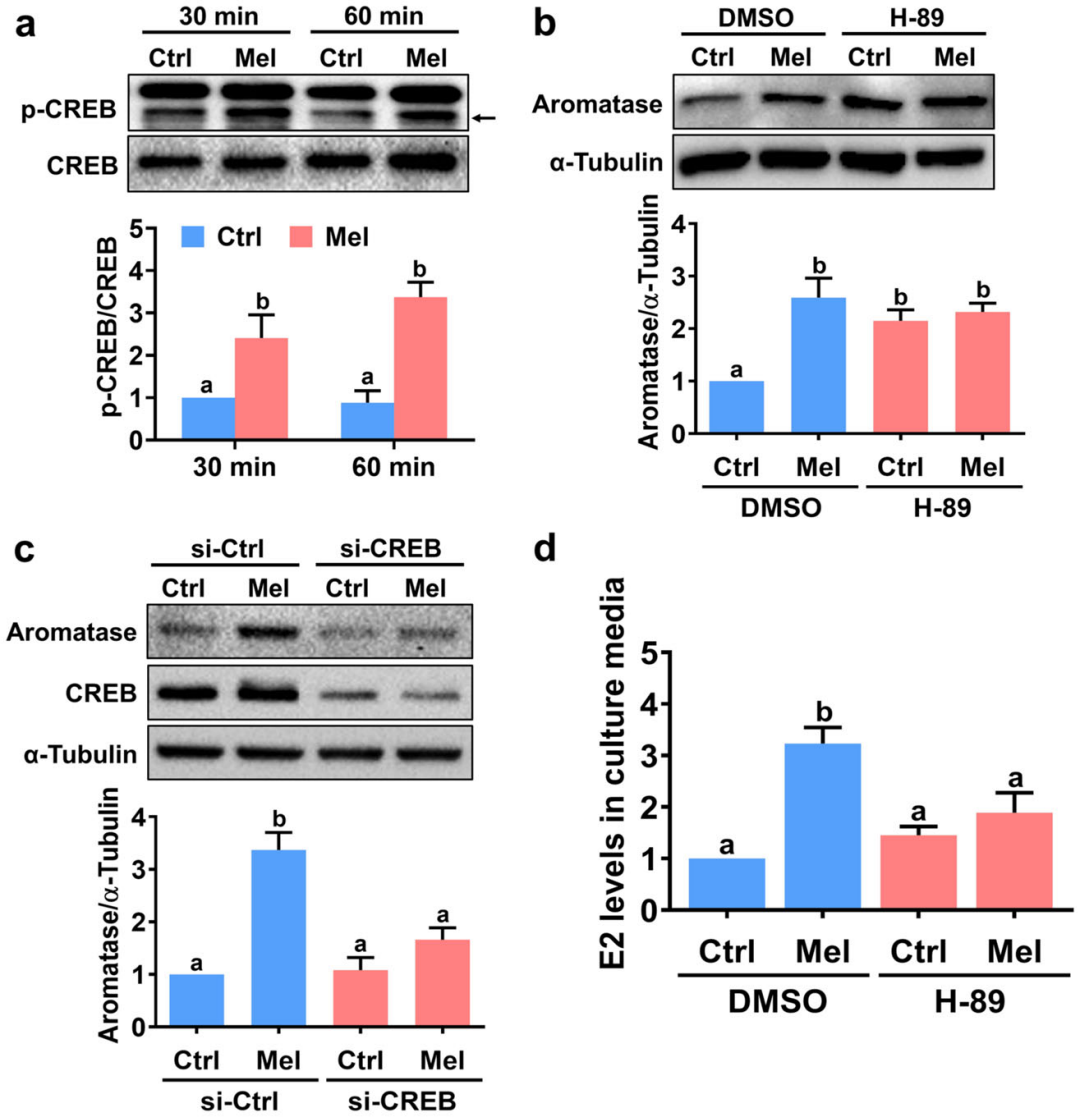
Melatonin-stimulated aromatase expression is mediated by the PKA-CREB pathway in primary hGL cells. **a** Cells were treated with 500 µM melatonin for 30 or 60 min, and the expression of both phosphorylated and total CREB was determined by western blotting. **b** Cells were pretreated with vehicle control (DMSO) or 10 µM H-89 for 30 min and then exposed to 500 µM melatonin for 24 h. Aromatase protein levels were examined by western blotting. **c** Cells were transfected with 100 nM control siRNA (si-Ctrl) or CREB siRNA (si-CREB) for 48 h and then treated with 500 µM melatonin for 24 h. Aromatase protein levels were examined by western blotting. **d** Cells were pretreated with vehicle control (DMSO) or 10 µM H-89 for 30 min and then exposed to 500 µM melatonin for 24 h. E2 levels in culture media were examined by ELISA. The results are expressed as the mean ± SEM of at least three independent experiments. Values without a common letter are significantly different (*p* < 0.05).

The protein kinase A (PKA)-cAMP-response element binding protein (CREB) pathway is the best known pathway that mediates aromatase gene expression in the ovary^25^. Therefore, we examined the effect of melatonin on the activity of CREB in hGL cells. As shown in Fig. 3a, melatonin treatment for 30 and 60 min increased phospho-CREB levels, indicating induction of CREB activation. The stimulatory effect of melatonin on aromatase protein levels was blocked by pretreatment with a potent selective inhibitor of PKA, H-89 (Fig. 3b). To prevent any possible nonspecific effects of the pharmacological inhibitor, endogenous CREB was knocked down by transfecting hGL cells with a specific siRNA targeting CREB. As shown in Fig. 3c, CREB siRNA significantly downregulated CREB protein levels in hGL cells, and the melatonin-induced upregulation of aromatase protein levels was attenuated by the knockdown of CREB. In addition, inhibition of PKA abolished melatonin-stimulated E2 production in hGL cells (Fig. 3d).

### Blocking melatonin function attenuates the pathogenesis of OHSS in mice

**Fig. 4:**
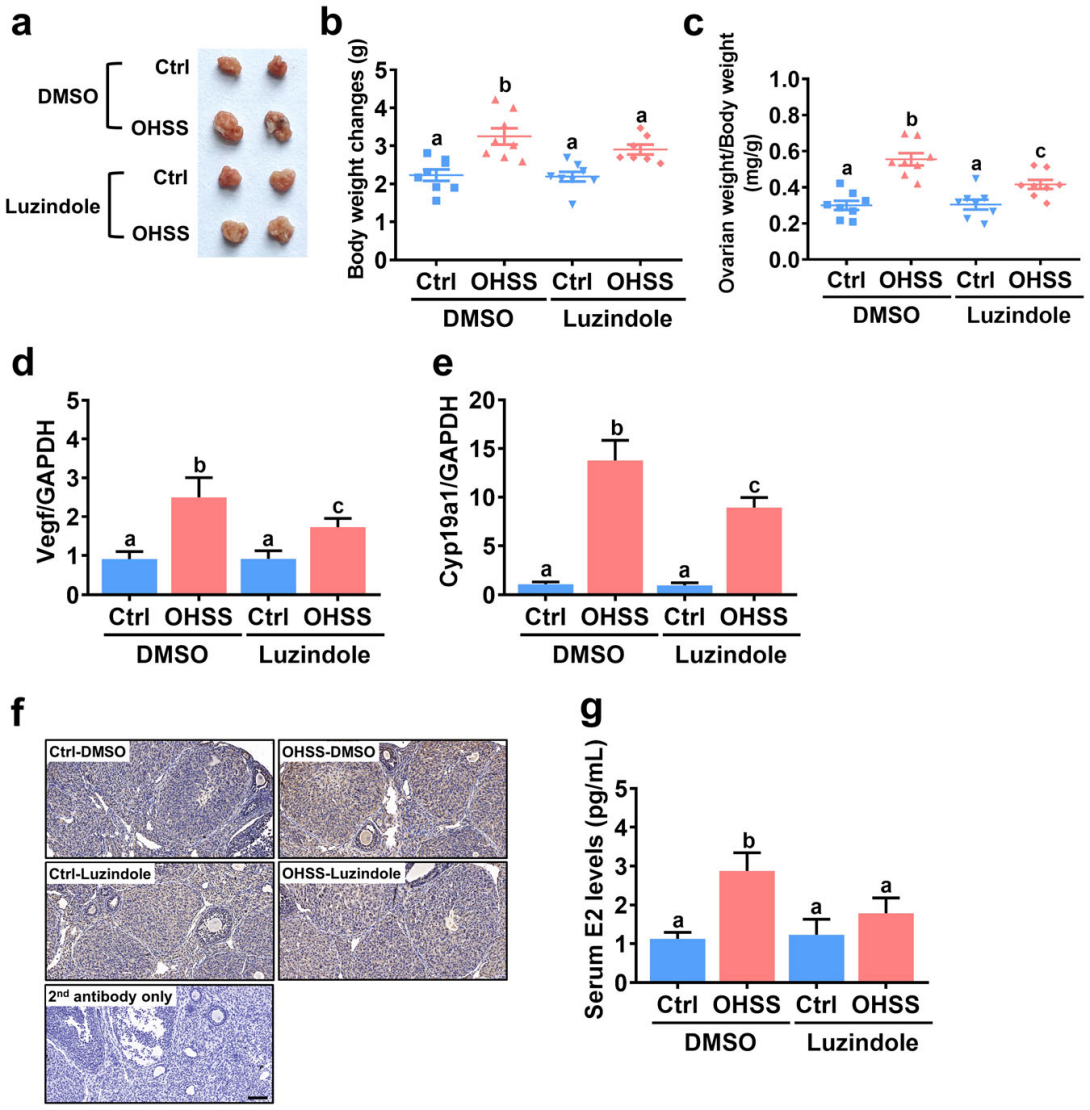
Blocking melatonin signaling inhibits the development of OHSS. **a** Representative ovaries of mice from each group were photographed. **b** Changes in body weight throughout the treatment period were evaluated after the mice were euthanized. **c** Ovarian weight relative to body weight was analyzed after the mice were euthanized. **d**, **e** The mRNA levels of VEGF (**d**) and aromatase (Cyp19a1) (**e**) in mouse ovaries were examined by RT-qPCR. **f** Representative images of immunostaining for aromatase in ovarian sections. Original magnification: ×200. The scale bar represents 50 μm. **g** The serum E2 levels in mice were examined by ELISA. The results are expressed as the mean ± SEM of at least three independent experiments. Values without a common letter are significantly different (*p* < 0.05).

To further explore the role of melatonin in regulating the pathogenesis of OHSS, we used luzindole to block the function of melatonin in a mouse model of OHSS. Consistent with previous studies, induction of OHSS significantly increased the size of the ovary, body weight, and ovarian weight in mice^26,27^. Blocking the function of melatonin alleviated the severity of the symptoms in the OHSS group, including increased size of the ovary, body weight, and ovarian weight (Fig. 4a–c). In addition, the mRNA levels of vascular endothelial growth factor (VEGF), a well-known marker for OHSS, were upregulated in the ovaries of OHSS mice compared to those of control mice, but the induction of VEGF mRNA levels was attenuated by the administration of luzindole (Fig. 4d). Interestingly, RT-qPCR and immunohistochemical analysis showed that the mRNA and protein levels of aromatase were significantly upregulated in the ovaries of OHSS mice compared to those of control mice. Importantly, the upregulation of aromatase expression levels was attenuated by inhibition the function of melatonin (Fig. 4e, f). Given the stimulatory effect of OHSS induction on aromatase expression in mouse ovaries, as expected, serum E2 levels were significantly increased in OHSS mice compared to control mice, and these increases were inhibited by the administration of luzindole (Fig. 4g). Taken together, these results reveal the important roles of melatonin and aromatase in the pathogenesis of OHSS.

### Aromatase expression levels are upregulated in hGL cells and follicular fluid from OHSS patients

**Fig. 5:**
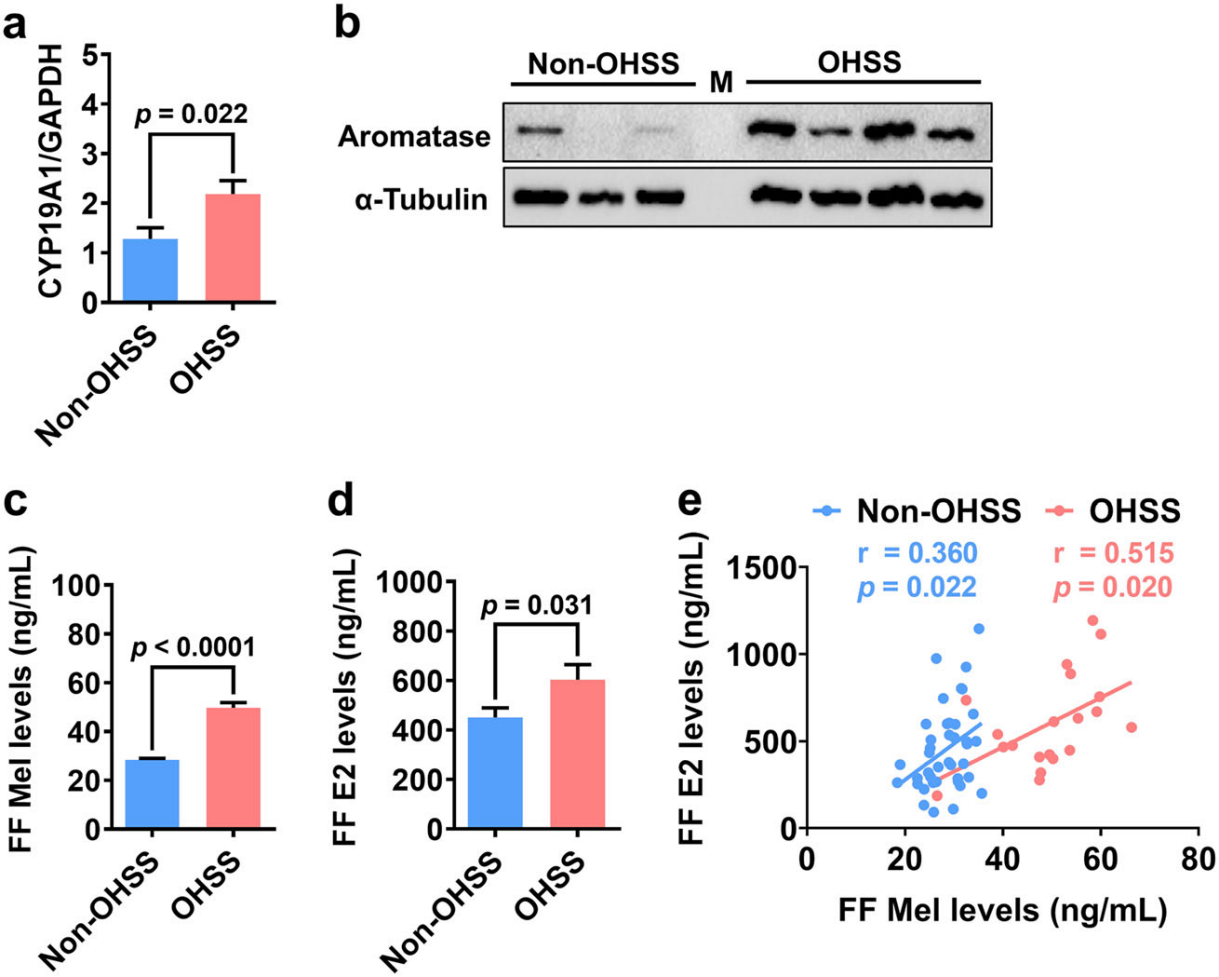
Aromatase expression and melatonin and E2 levels in the follicular fluid are increased in OHSS patients. **a** Aromatase mRNA levels (CYP19A1) in hGL cells from non-OHSS (*n* = 8) and OHSS patients (*n* = 8) were examined by RT-qPCR. **b** Aromatase protein levels in hGL cells from non-OHSS (*n* = 3) and OHSS patients (*n* = 4) were examined by western blotting. M: protein marker. **c**, **d** Melatonin (**c**) and E2 (**d**) levels in the follicular fluid of non-OHSS (*n* = 40) and OHSS (*n* = 20) patients were examined by ELISA. **e** Pearson’s correlation analysis was performed to evaluate the correlation between two values. Melatonin levels were positively correlated with E2 levels in the follicular fluid of non-OHSS (*n* = 40) and OHSS patients (*n* = 20). The results are expressed as the mean ± SEM. Values without a common letter are significantly different (*p* < 0.05).

We next examined the expression of aromatase in hGL cells from non-OHSS and OHSS patients. As shown in Fig. 5a, aromatase mRNA levels were higher in hGL cells from OHSS patients than in those from non-OHSS patients. Western blotting showed similar results; aromatase protein levels were upregulated in hGL cells from OHSS patients compared to those from non-OHSS patients (Fig. 5b). Consistent with our previous study^15^, melatonin levels were significantly higher in the follicular fluid of OHSS patients than in that of non-OHSS patients (Fig. 5c). As expected, E2 levels were considerably higher in the follicular fluid of OHSS patients than in that of non-OHSS patients (Fig. 5d). Interestingly, Pearson’s correlation analysis showed that the expression levels of melatonin and E2 in the follicular fluid of non-OHSS and OHSS patients were positively correlated (Fig. 5e).

## Discussion

In the present study, we show that treatment with melatonin stimulates aromatase expression and E2 production by activating the PKA-CREB pathway in hGL cells. In addition, we demonstrate that the expression levels of aromatase and serum E2 are upregulated in a mouse model of OHSS compared to control mice. Inhibition of melatonin function attenuates the development of OHSS and upregulations of aromatase and serum E2 levels. Moreover, our clinical results reveal that aromatase expression levels are upregulated in hGL cells from OHSS patients. The levels of melatonin and E2 in follicular fluid are positively correlated, and the levels of both hormones are significantly higher in OHSS patients than in non-OHSS patients. Taken together, these results indicate that the elevated levels of melatonin in follicular fluid contributes to the high E2 levels in OHSS by upregulating aromatase expression.

The stimulatory effect of melatonin on E2 production in granulosa cells has been reported previously^18–20^. However, whether melatonin stimulates E2 production in granulosa cells by upregulating aromatase expression remains unknown. Our study showed that treatment of hGL cells with melatonin upregulates aromatase expression, which consequently contributes to increased E2 production. It is interesting to note that melatonin has been shown to exert an oncostatic effect in human breast cancer cells by downregulating aromatase expression and activity^28^. The human aromatase gene *CYP19A1* contains 10 exons. The aromatase protein is encoded by exons II-X. Upstream of exon II are a number of alternative first exons that encode unique 5’-untranslated regions of human *CYP19A1* in a tissue-specific fashion. Therefore, while the aromatase proteins that are encoded in different tissues are identical, the aromatase gene is driven by a tissue-specific promoter that lies upstream of different first exons in different tissues. To date, 10 alternative tissue-specific promoters have been identified in the human *CYP19A1* gene^29^. The ovarian *CYP19A1* gene contains promoter II, and its expression is driven by this gonad-specific promoter^30^. In breast cancer, a distinct set of aromatase promoters, namely, I.3, I.7, and II, is activated, which leads to increases in aromatase expression and E2 production in tumor cells and breast adipose tissue adjacent to tumors^31^. This special gene structure could explain why the regulation of aromatase expression occurs in a tissue-specific manner.

It is well known that cAMP-PKA is the main intracellular messenger mediating FSH-stimulated aromatase expression in granulosa cells^3^. CREB is a transcription factor that can be phosphorylated and activated by PKA upon FSH treatment. Activated CREB binds to the cAMP-response element (CRE) in the *CYP19A1* promoter and regulates its expression^32,33^. Overexpression of mutant CREB blocks FSH-induced E2 production in primary cultured rat granulosa cells^34^. These studies demonstrate that CREB activation is required for the expression of aromatase in granulosa cells. Consistent with previous studies in other cell types^35,36^, our study showed that CREB is activated by melatonin treatment in hGL cells. In addition, our results reveal that activation of the PKA-CREB pathway is required for melatonin-stimulated aromatase expression and E2 production. The mRNA levels of CREB are significantly higher in breast adipose tissue than in normal breast adipose tissue^37^. Interestingly, a recent study reported that the expression levels of CREB are upregulated in induced pluripotent stem cell (iPSC)-derived granulosa cells and adult granulosa cells from PCOS patients compared with those from non-PCOS patients^38^. Whether the expression levels of CREB varies in granulosa cells from OHSS patients remains unknown and needs to be examined.

OHSS is characterized by enlargement of the ovaries and a fluid shift from the intravascular space to the third space due to increased capillary permeability and angiogenesis^5^. Decreasing E2 levels helps to prevent the development of OHSS^39^. However, to date, the role of elevated E2 levels in OHSS remains obscure. VEGF, a well-known marker for OHSS pathology, has been identified as a major vasoactive factor and angiogenic factor in OHSS^40^. Our previous study revealed that melatonin treatment upregulates VEGF expression in hGL cells^15^. In addition, a cAMP-activated chloride channel, cystic fibrosis transmembrane conductance regulator (CFTR), is upregulated by E2 and contributes to the pathogenesis of OHSS^41^. CFTR plays important roles in the regulation of the secretion and absorption of salt and fluid^42^. Taken together, these studies suggest that elevated E2 levels could contribute to the pathogenesis of OHSS by increasing capillary permeability, which influences fluid shift^43^. Interestingly, CFTR is expressed in human granulosa cells and mediates FSH-induced aromatase expression and E2 production^44^. Therefore, future studies are needed to examine whether the expression of CFTR can be regulated by melatonin in human granulosa cells. In addition, whether CFTR expression varies in granulosa cells from OHSS patients remains unknown and warrants further investigation.

Melatonin and melatonergic drugs have been used to treat insomnia and circadian rhythm disorders^45^. Increasing evidence has demonstrated that, in various cancers, the oncostatic effects of melatonin can be achieved through changes in cell proliferation, apoptosis, metabolism, migration, invasion, and angiogenesis induced by receptor-mediated signaling and by reactive oxygen and nitrogen species scavenging induced by receptor-independent actions. Melatonin has been shown to enhance the therapeutic effect of various anticancer drugs in clinical trials and might help improve the sleep and life quality of cancer patients^46^. Previous animal studies have revealed the antidepressant-like effect of luzindole and the effect of another melatonin receptor antagonist, S22153, on circadian rhythm entrainment^47,48^. However, these effects of melatonin receptor antagonists have only been evaluated in preclinical studies. Therefore, future studies are required to determine the therapeutic potential of melatonin receptor antagonists.

In summary, the present study demonstrates that treatment with melatonin upregulates aromatase expression in hGL cells, resulting in increased E2 production. These effects are mediated by MT1 and MT2 receptors and the PKA-CREB pathway. Blocking the function of melatonin inhibits the pathogenesis of OHSS in a mouse model of OHSS. In addition, we show that the expression of aromatase in ovaries and serum E2 levels are upregulated in OHSS mice. Importantly, the upregulation of aromatase and serum E2 levels is attenuated by inhibition of melatonin signaling. Clinically, aromatase expression levels are upregulated in hGL cells from OHSS patients. Melatonin and E2 levels in follicular fluid are significantly higher in OHSS patients than in non-OHSS patients. Moreover, melatonin levels are positively correlated with E2 levels in follicular fluid. Taken together, these results suggest a role for melatonin in the regulation of aromatase expression and E2 production in hGL cells and provide a potential mechanism through which melatonin contributes to the high E2 levels in OHSS. The findings of our study could lead to the development of alternative therapeutic approaches for OHSS.

## Supplementary information

Supplemental Table 1
