# Supplementary material for: Melatonin stimulates aromatase expression and estradiol production in human granulosa-lutein cells: relevance for high serum estradiol levels in patients with ovarian hyperstimulation syndrome
Source: Exp Mol Med. 2020 Aug 27;52(8):1341–50. doi: 10.1038/s12276-020-00491-w (PMC8080626; doi:10.1038/s12276-020-00491-w)
Supplement: Supplementary file 1 — Supplemental Table 1 [file 12276_2020_491_MOESM1_ESM.pdf]

Supplemental Table 1. Characteristics of Non-OHSS and OHSS patients

	Non-OHSS group (n=40) Mean ± SEM	OHSS group (n=20) Mean ± SEM	*p*
Age	28.65 ± 0.53	29.37 ± 0.37	0.285
BMI	22.93 ± 0.37	22.79 ± 0.41	0.787
AFC	14.29 ± 0.69	17.98 ± 0.74	0.0004
Basal serum FSH (mIU/ml)	6.69 ± 0.19	6.27 ± 0.17	0.121
Basal serum LH (mIU/ml)	4.80 ± 0.30	7.65 ± 0.86	0.0013
Basal serum E2 (pg/ml)	30.61 ± 2.90	32.53 ± 2.44	0.618
Basal serum P4 (ng/ml)	0.57 ± 0.04	0.52 ± 0.03	0.4372
Basal serum PRL (ng/ml)	18.42 ± 1.08	18.83 ± 1.07	0.7881
Total Gn dose (Ampoules)	1691 ± 75.50	1549 ± 71.29	0.177
E2 in serum on hCG administration day	4053 ± 337.5	7941 ± 387.7	<0.0001
The number of oocytes retrieved	11.44 ± 0.75	20.10 ± 0.96	<0.0001
